# Supplementary material for: Integrating computational chemistry and machine learning to predict KRAS mutation-induced resistance
Source: bioRxiv. 2026 Apr 14:2026.04.10.717640. Originally published 2026 Apr 11. Preprint. [Version 2] doi: 10.64898/2026.04.10.717640 (PMC13081811; doi:10.64898/2026.04.10.717640)
Supplement: Supplement 1 [file NIHPP2026.04.10.717640v2-supplement-1.pdf]

bioRxiv preprint doi: https://doi.org/10.64898/2026.04.10.717640; this version posted April 14, 2026. The copyright holder for this preprint (which was not certified by peer review) is the author/funder, who has granted bioRxiv a license to display the preprint in perpetuity. It is made available under aCC-BY 4.0 International license.

Supporting Information

S1 Table. XGBoost model evaluation.

Run / Metric	Accuracy	Precision	Recall	AUC	Specificity
1	0.923	0.857	1.000	0.988	0.857
2	1.000	1.000	1.000	1.000	1.000
3	0.962	0.917	1.000	0.976	0.933
4	0.923	0.909	0.909	0.994	0.933
5	0.769	0.667	0.909	0.903	0.667
6	0.885	0.867	0.929	0.958	0.833
7	0.885	1.000	0.813	0.994	1.000
8	0.962	0.929	1.000	0.976	0.923
9	0.962	0.917	1.000	1.000	0.933
10	0.923	0.882	1.000	0.939	0.818
Average ±SD	0.919±0.064	0.894±0.094	0.956±0.064	0.973±0.031	0.890±0.100

Run / Metric	Accuracy	Precision	Recall	AUC	Specificity
1	0.962	0.929	1.000	0.994	0.923
2	0.731	0.667	0.923	0.947	0.538
3	0.923	0.867	1.000	0.988	0.846
4	0.846	0.909	0.769	0.941	0.923
5	0.923	0.923	0.923	0.970	0.923
6	0.923	0.867	1.000	0.982	0.846
7	0.846	0.765	1.000	0.953	0.692
8	1.000	1.000	1.000	1.000	1.000
9	0.962	1.000	0.923	0.953	1.000
10	0.962	0.929	1.000	1.000	0.923
Average ±SD	0.908±0.079	0.885±0.103	0.954±0.074	0.973±0.023	0.862±0.144

S2 Table. PLS-DA model evaluation.

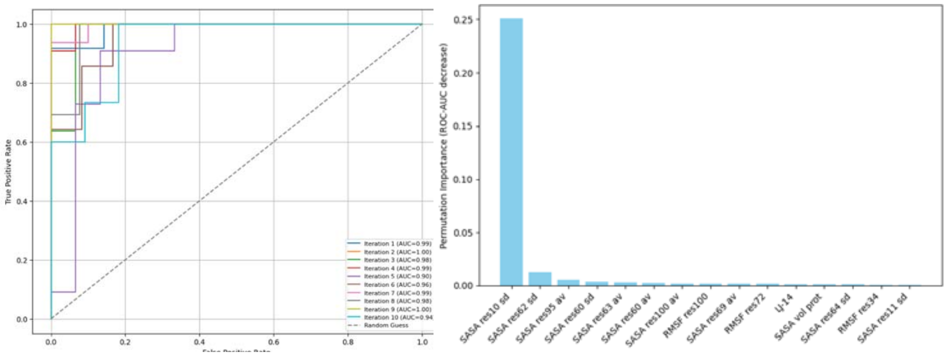

S1 Fig. XGBoost model performance. Left: XGBoost ROC-AUC across 10 iterations. **Right**: top 15 Feature Importance averaged over 10 iterations calculated using a Permutation technique. This model, even after small-data set adjustment, still relies on one dominant feature SASA (SD) of G10. It agrees with the models studied in the publication.

bioRxiv preprint doi: https://doi.org/10.64898/2026.04.10.717640; this version posted April 14, 2026. The copyright holder for this preprint (which was not certified by peer review) is the author/funder, who has granted bioRxiv a license to display the preprint in perpetuity. It is made available under aCC-BY 4.0 International license.

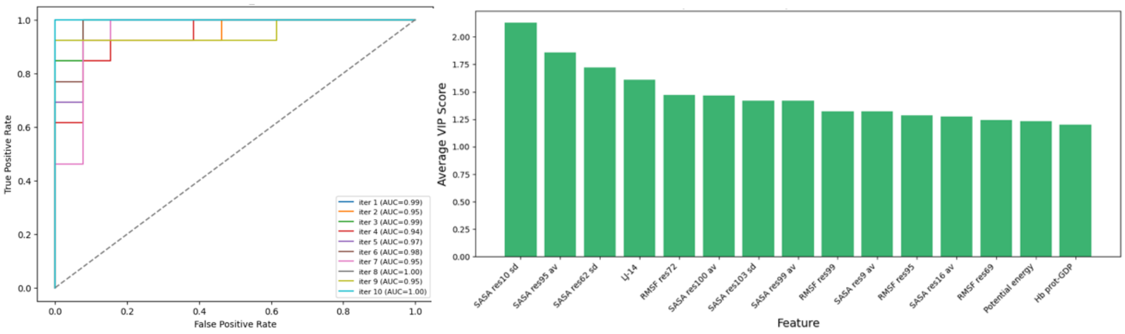

S2 Fig. PLS-DA model performance. **Left**: PLS-DA ROC-AUC across 10 iterations. **Right**: top 15 Feature Importance averaged over 10 iterations calculated using a Variable Importance in Projection (VIP) scores, which estimate each feature contribution to the production. This model does not rely heavily on a few features, but consensus between several equally important. The result agrees with the models studied in the publication.

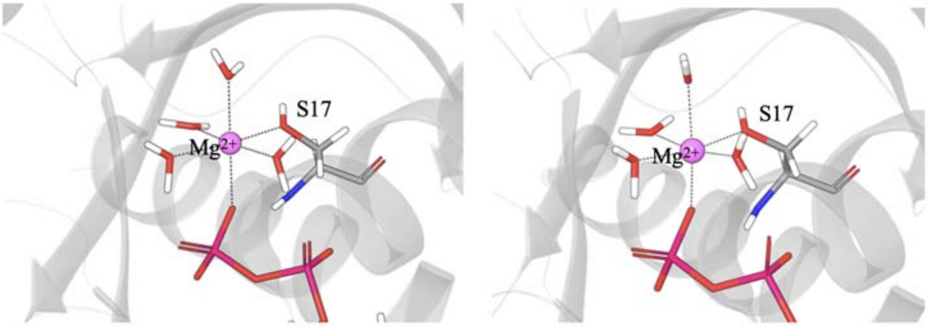

S3 Fig. Water shell around Mg^{2+}.
Left: initial structure (PDB ID: 6OIM, X-ray).
Right: system's starting structure after the NPT equilibration ready for the simulation run.
